# Perfluorinated Probes for Noncovalent Protein Recognition and Isolation

**DOI:** 10.1021/acs.bioconjchem.9b00846

**Published:** 2020-01-11

**Authors:** Ivan Bassanini, Corinna Galli, Erica E. Ferrandi, Fabiana Vallone, Annapaola Andolfo, Sergio Romeo

**Affiliations:** † Dipartimento di Scienze Farmaceutiche, Università degli Studi di Milano, 20133 Milano, Italy; ‡ Istituto di Chimica del Riconoscimento Molecolare - Consiglio Nazionale delle Ricerche, 20131 Milano, Italy; § ProMiFa, Protein Microsequencing Facility, Ospedale San Raffaele, 20132 Milano, Italy

## Abstract

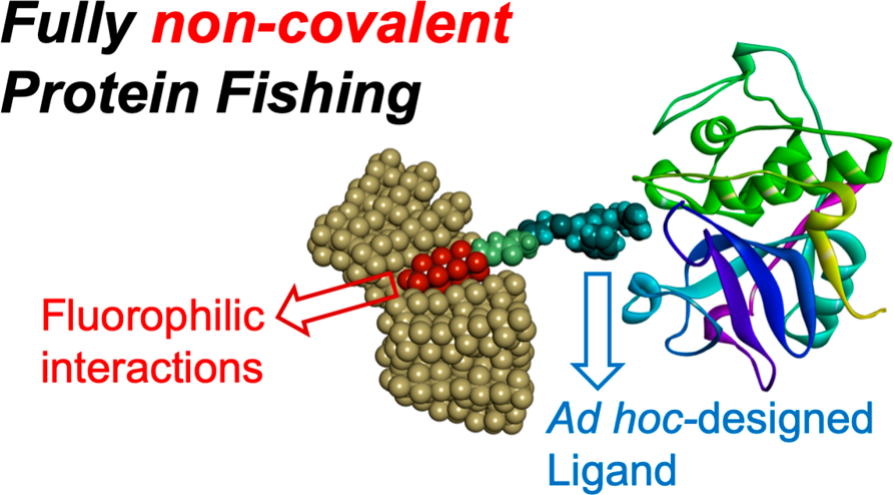

Perfluorinated organic compounds (PFCs) are nontoxic, biocompatible, bioavailable, and bioorthogonal species which possess the unique ability to segregate away from both polar and nonpolar solvents producing a compact fluorophilic phase. Traditional techniques of fluorous chemical proteomics are generally applied to enrich biological samples in target protein(s) exploiting this property of PFCs to build fluorinated probes able to covalently bind to protein ensembles and being selectively extracted by fluorophilic solvents. Aiming at building a strategy able to avoid irreversible modification of the analyzed biosystem, a novel fully noncovalent probe is presented as an enabling tool for the recognition and isolation of biological protein(s). In our strategy, both the fluorophilic extraction and the biorecognition of a selected protein successfully occur via the establishment of reversible but selective interactions.

When fluorine, the most electronegative element in the periodic table, substitutes hydrogen atoms in the framework of a bioactive organic molecule, its unique chemical properties deeply affect the physiochemical characteristics of the original hydrocarbon species,^1,2^ e.g., by improving its metabolic stability,^3,4^ bioavailability,^5,6^ and binding affinity.^7^ Perfluorinated organic compounds (PFCs), characterized by the complete substitution of a *CmHn* framework with a *CmFn* chain, are nontoxic,^8,9^ biocompatible, bioavailable, and bioorthogonal^10^ species largely exploited *in vivo* as enabling tools for innovative supramolecular bioapplications.^11−14^ As an example, PFCs offer the possibility of acquiring *in vivo* imaging data through the application of ^19^F-magnetic resonance spectroscopy (MRS) and ^19^F magnetic resonance imaging (MRI).^15−20^

Interestingly, PFCs also possess the ability to segregate away from both polar and nonpolar solvents, producing a compact separated fluorophilic phase, i.e., the fluorous phase.^13,21^ This peculiar feature allows a wide number of biological applications ranging from their exploitation as fluorosurfactants^8,22−25^ in the field of nanotechnological drug delivery^14,26−30^ to fluorous solid phase extraction (F-SPE)^31^ to be applied, e.g., in the development of innovative strategies of fluorous chemical proteomics.^10,32−34^

In general, fluorous-based chemical proteomics is employed to enrich samples of biological origin in specific peptide subsets and relays on the use of *ad hoc* designed perfluorinated probes^35−39^ to extract target protein(s), e.g., from cell lysates or crude biological samples. Biotin/avidin- or streptavidin-based systems are noteworthy examples of this strategy in which fluorolabeled biotin interacts and “tags” protein via chemical reactions with the ε-amino group of lysine residues allowing the selective extraction and recognition of tagged proteins.^40−44^ While the fluorophilic interactions involved in F-SPE are, by definition, noncovalent, the mechanisms of action of the fluoro-labeled probes employed in “classical” fluorous proteomics are mainly based on the establishment of a strong covalent bond with their biomolecular target(s). This kind of approach, even if successful, is not free from drawbacks. In fact, the building of a covalent bond with biological entities necessarily produces an irreversible modification of the analyzed system that, combined with the common use of cell lysates, precludes both the survival of the biological system (*de facto* preventing the possibility of conducting multiple experiments) and harms the integrity of the collected data. Moreover, the selectivity of biotin/avidin-based systems can often be impaired by the aspecific biorecognition of undesired protein ensembles, thus obstructing the possibility of building selective systems to study the specific pattern of interactions of a bioactive molecule, i.e., the interactome of a drug.

With the aim of merging the possibility of conducting in-line analysis (MRI or MRS) and F-SPEs while conveniently interacting with selected biological protein-target(s) through a network of noncovalent bonds established with specific hot-spot residues, we designed and built perfluorinated, peptide-conjugated probes for protein(s) recognition and isolation (Figure 1). The design of our novel probes was based on the identification of three distinct regions: a perfluorinated alkyl chain acting as fluorophilic tag, a bioactive portion able to reversively interact with selected hot-spots in protein target(s), and a linking region between the two listed cores.

**Figure 1:**
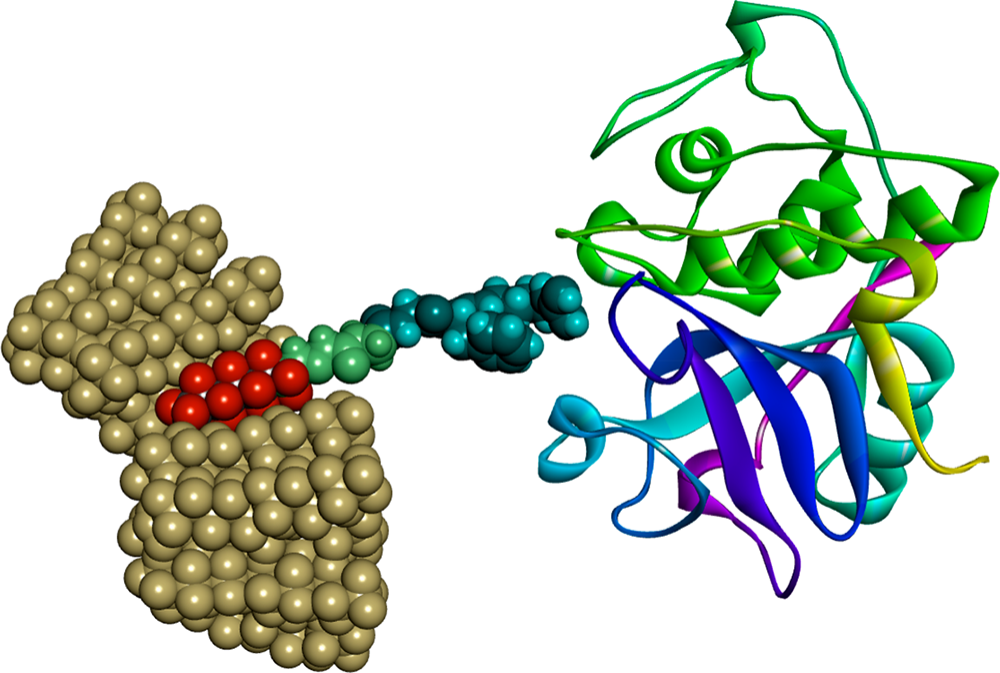
Representation of the noncovalent interaction between fluoro-labeled probe and its protein target. The yellow-gray layer of PFCs is depicted in the act of surrounding the fluorophilic portion (red dots) of the probe which is interacting with papain through its peptide bioactive portion (cyan dots) connected to the fluorous-tag via a linking region (green).

A perfluorinated medium-length C_7_F_15_ alkyl chain was selected as fluorophilic tag. Starting from commercially available 2,2,3,3,4,4,5,5,6,6,7,7,8,8,8-pentadecafluorooctan-1-ol, our first building block (A) was obtained via a straightforward derivatization with α-bromoacetic acid^45^ (see Supporting Information). The free carboxylic acid group was inserted to allow further derivatization by simple peptide coupling chemistry, while the ether bond served as spacer between the fluorophilic moiety and the still to be implanted bioactive portion (Figure 2). As a proof of concept, we decided to use papain, a well characterized cysteine protease (MW = 23 kDa, EC number: 3.4.22.2) studied, e.g., as a template for the structure-based design of Cathepsin K inhibitors,^46^ as model protein. As reported in the literature, papain is prone to inactivation by the covalent modification of the cysteine residues characterizing its active site mediated by diazomethyl ketones.^47,48^

**Figure 2:**
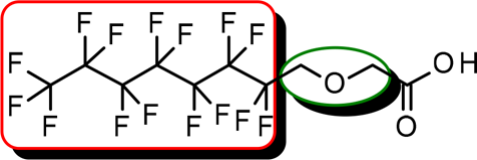
Structure of the common fluorophilic building block (A).

Moreover, and most importantly to us, the tetrapeptide GGYR^49^ was described as a noncovalent active-site ligand successfully applied in ionic-strength dependent affinity chromatography experiments.

Thus, two perfluorinated papain-interacting probes (**1** and **2**, Figure 3) were designed and synthesized (for synthetic details, see Supporting Information) to be applied in two different experiments of papain recognition and isolation via F-SPEs. The performances of probes **1** and **2** as active site inhibitors were verified by running a spectrophotometric assay following literature procedures^47−49^ (for details, see Supporting Information).

**Figure 3:**
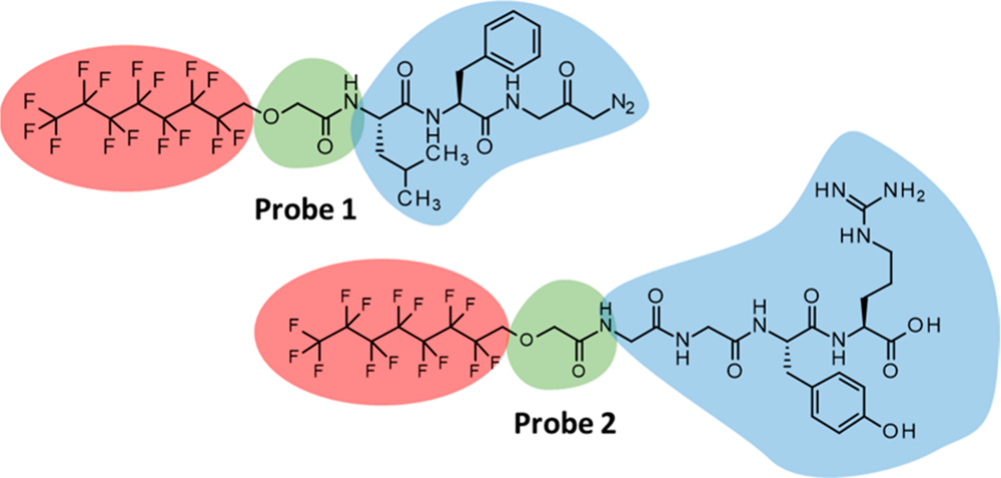
Structures of perfluorinated probes **1** and **2**.

A F-SPE routine experiment was built (Figure 4). Briefly, a commercially available preparation of papain from *Carica papaya* (known to be fraught with protein contaminations) was chemically activated and incubated in the presence of one of the two fluorophilic probes. After that, the F-SPE was run using a simple Teflon cartridge loaded with a C_8_ reverse phase perfluorinated resin (C8–F resin) collecting fractions using a “fluorophilic gradient”. The diazomethyl ketone-based probe (**1**) was prepared and used to validate the described experimental workflow following a traditional covalent approach to isolate papain. Control experiments were designed and conducted as well.

**Figure 4:**
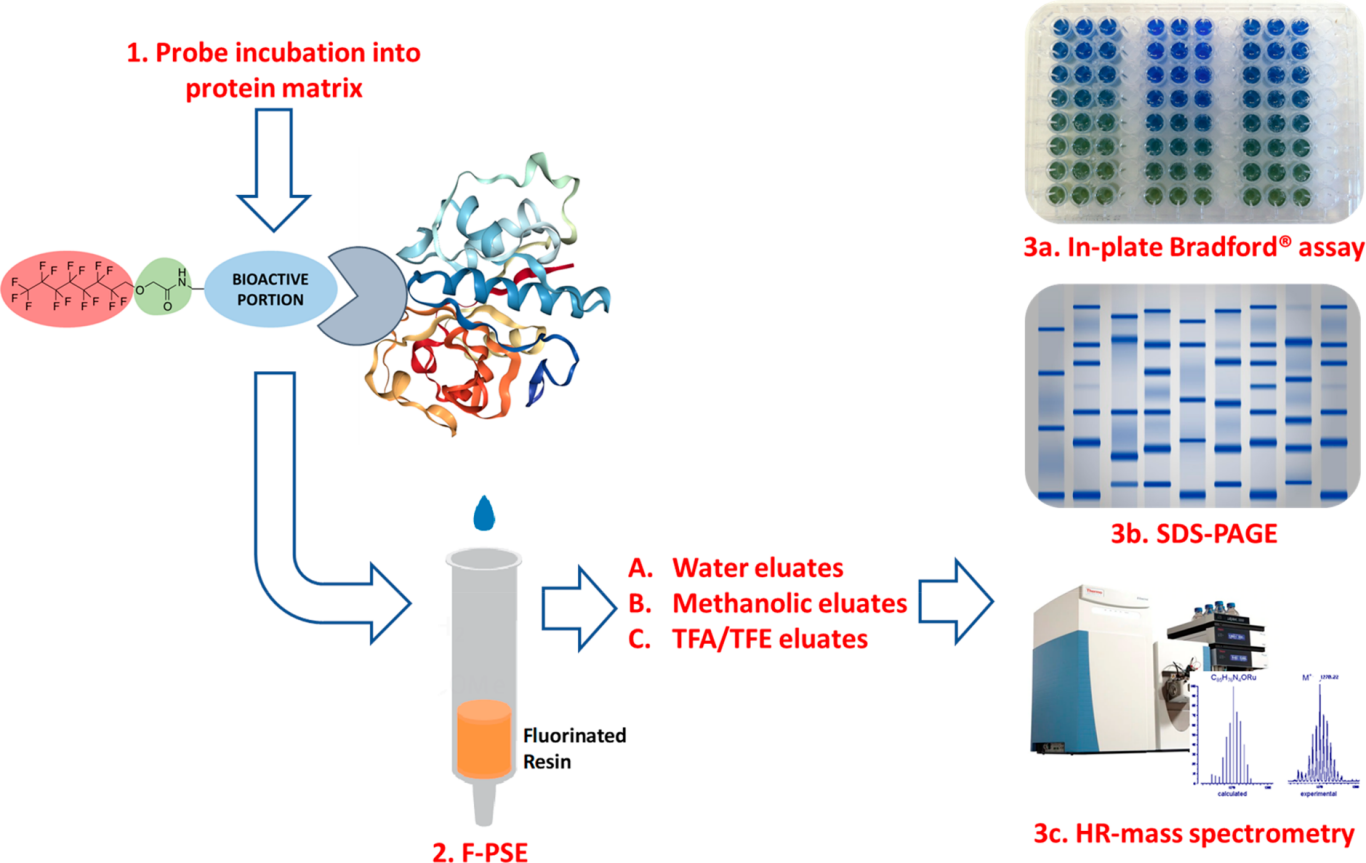
Experimental workflow for protein recognition and isolation.

At first, a reproducible protocol for papain activation and fluorous tagging was built. Specifically, the protein (6.7 mg, 145 μM) was dissolved in PB-buffer (85 mM, pH 6) and incubated in the presence of β-mercaptoethanol (21 mM) and EDTA (9 mM) for 45 min at 25 °C. Freshly activated papain was then incubated with an excess of the perfluorinated probe (40 mg, 5 mM, 15 min, 15 °C) allowing its binding to the inhibitory peptide sequences.

Given the peculiar wettability properties of perfluorinated resins, papain loading onto the C8–F resin from the mentioned aqueous solution needed to be carefully investigated. Water is indeed a highly fluorophobic medium which does not allow the establishment of proper interactions between dissolved tagged proteins and the perfluorinated beads. To overcome this issue, we needed to increase the fluorophilic character of the papain aqueous solutions we were working with by adding a proper amount of a fluorophilic solvent, i.e., methanol.

Thus, papain conformational stability toward methanol, a crucial issue to be assessed for the development of our “*fishing*” method, was investigated via CD-analysis (as reported in the Supporting Information). Papain retained its native folding in the presence of the 25% v/v of methanol and was found still partially folded when dissolved in a 50% v/v mixture of methanol and PB buffer. Therefore, a solution of freshly activated papain treated with probe **1** (4.4 mM) was incubated under gentle orbital shacking (50 rpm) for 5 min with the C8–F resin in the presence of methanol (25% v/v). After the loading step, we then proceeded with the elutions working in gradient of fluorophilicity. At first, we eluted with 0.5 CV of distilled water to remove the activating/loading solutions. Four CV were then used to theoretically wash from the resin all the water-soluble substances (e.g., buffer salts) and, if any was present, unbound papain. Fluorophilic elutions were then conducted using pure methanol as the mobile phase. Since proteins are known to tightly interact with solid supports to the point that the enzymatic adsorption onto resin beads can be done by simple “*salting-out strategies*” from concentrated solutions and given the outstanding stability of papain toward denaturing media, a strongly fluorophilic and acid elution (4 CV) was also conducted using a 20% v/v solution of trifluoracetic acid (TFA) in trifluoroethanol (TFE) to ensure that all the supported papain was properly eluted. The three different sets of collected eluates were concentrated *in vacuo* removing any traces of organic solvents. Residues were weighed, taken up with water (0.25 CV), and then analyzed by qualitative means (run in 250 μL 96-well plate), quantitative Bradford assays, SDS-PAGE, and, when necessary, high-resolution mass spectrometry.

Figure 5 summarizes the results obtained from the mentioned elution experiment. As can be seen both by the bright blue color of the in-plate Bradford assay (Figure 5A) and by the band detected at *ca*. 20 kDa in SDS-PAGE (Figure 5B), some unbound papain (*ca*. 2 mg) was recovered in the water eluates. Interestingly, according to our hypothesis regarding the possible establishment of tight protein–resin interactions, the subsequent methanolic eluates were found to be void of any protein materials. Instead, papain was successfully and selectively recovered (*ca*. 4 mg) in the highly fluorophilic and acidic eluates composed by the 20% v/v TFA in TFE (Figure 5A and B). High-resolution mass analysis (methods and obtained data are reported *in extenso* in Supporting Information) were conducted on the highly fluorophilic eluates to further confirm the nature of the isolated protein materials. According to what was highlighted by SDS-PAGE (Figure 5B), the 20 kDa band was successfully identified as a mixture of all the different isoforms of papain contained in the commercially available source used.

**Figure 5:**
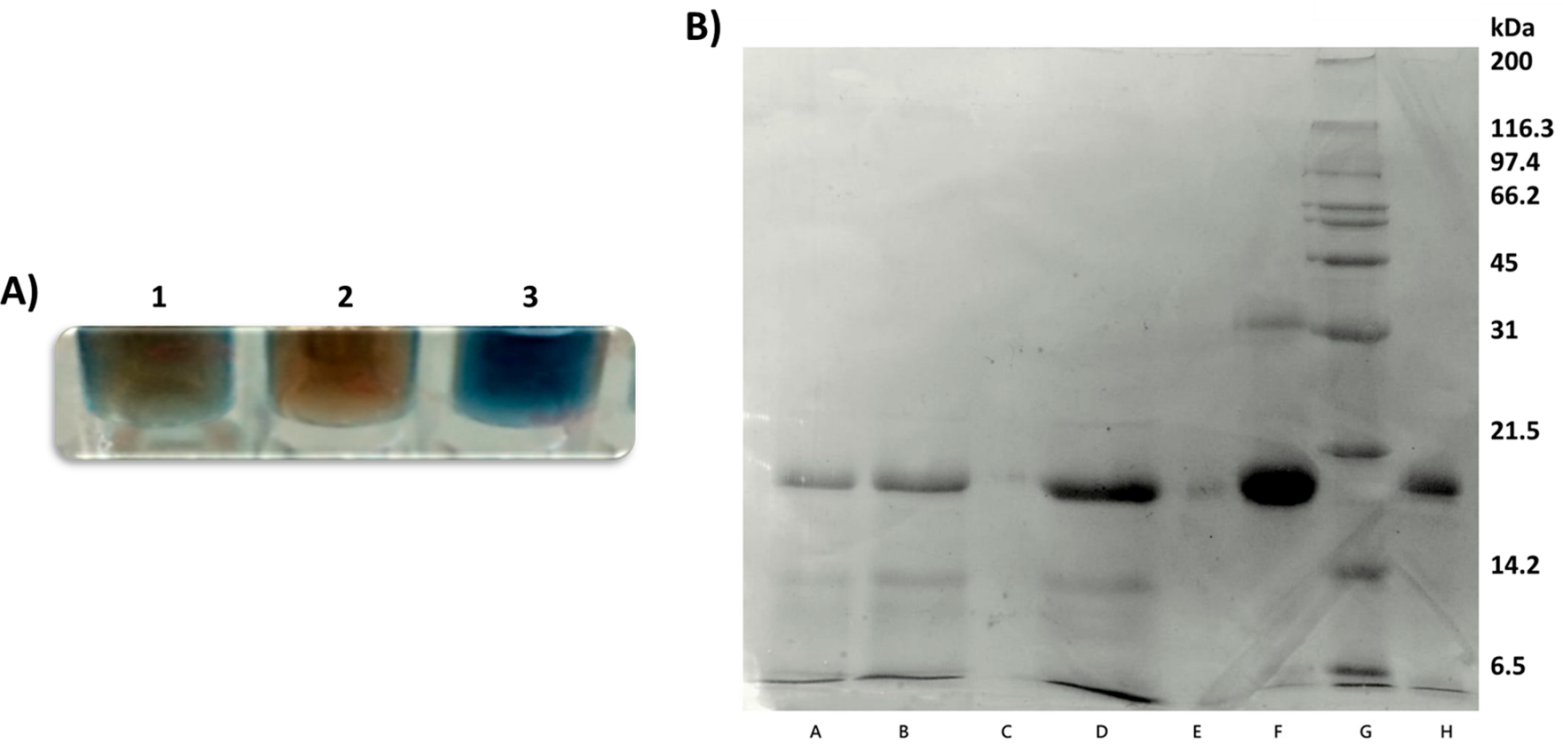
Results of the F-SPE in the presence of the covalent probe **1**. (A) Qualitative in-plate Bradford analysis: 1, water washings; 2, methanolic elution; 3, eluates collected in 20% TFA in TFE. (B) SDS-PAGE. Control experiment: A, water and B, methanolic washings; C, highly fluorophilic eluates. Fluorophilic papain isolation: D, water washings; E, methanolic eluates; F, highly fluorophilic eluates; G, MW markers; H, standard of papain.

Moreover, to confirm the selective, fluorophilic retention of the fluoro-labeled papain on the C8–F resin after washing, a control experiment was run by subjecting papain to the described process (the summarized method is reported in Supporting Information) but in the absence of probe **1**. All the loaded papain (*ca*. 6 mg) was washed from the C8–F resin during the first steps of water and methanolic washings. No protein was instead found in the highly fluorophilic TFA/TFE eluates as demonstrated by the SDS-PAGE presented in Figure 5B.

The experiments run using probe **1** allowed us to both validate the experimental workflow proposed (Figure 4) and gain some crucial information to build the consequent noncovalent protein isolation using probe **2**.

Specifically, the absence of protein in the methanolic eluates suggested to us how the simplified “fishing system” depicted in Figure 1 could be actually far from being the real and/or only operating mechanism at the basis of the observed selectivity. Despite remarkable papain stability toward methanol, in fact, it could be argued that the methanolic elutions should be fluorophilic and denaturing enough to fully recover all the papain bound to the resin in virtue of its covalently attached fluorous tag.

The experimental data collected, however, showed a different situation in which the majority of all the protein material loaded on the resin could be isolated only after an elution with a strong fluorophilic and acid media (20% v/v TFA in TFE). To the best of our knowledge, this evidence should suggest a more complex interacting mechanism between the fluoro-tagged protein(s) and the resin itself (Figure 6).

**Figure 6:**
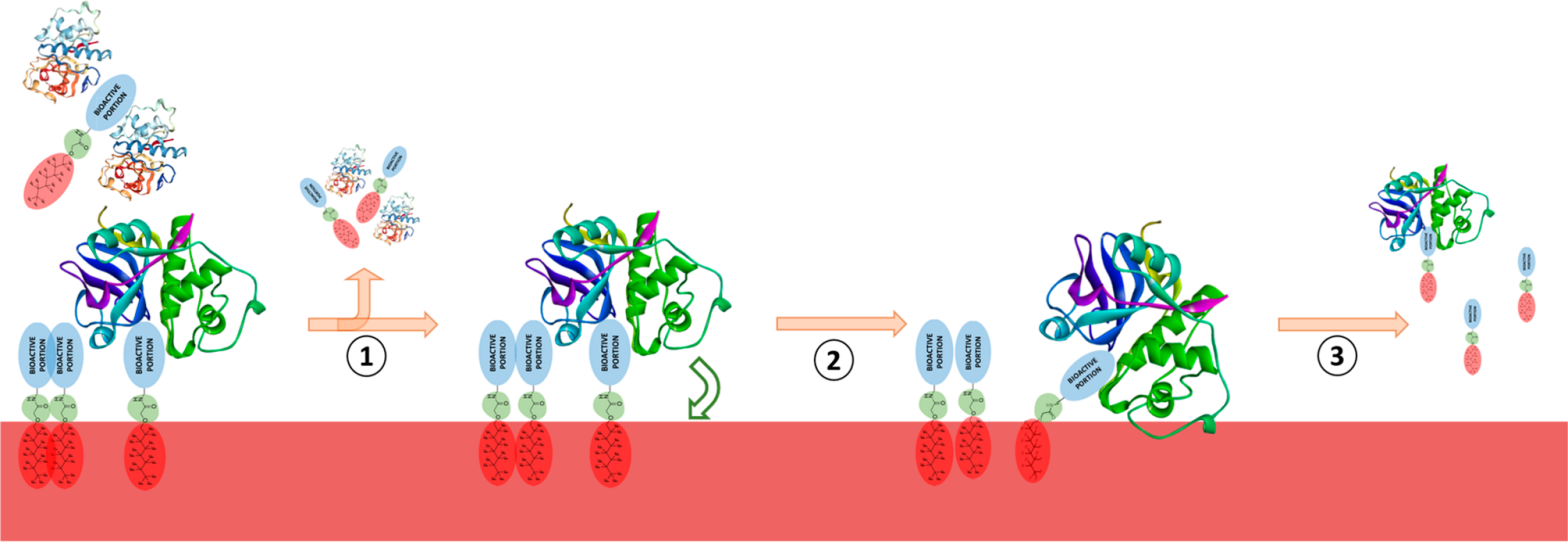
Schematic representation of the hypothesized papain adsorption onto the C8–F resin. (1) Tagged papain is loaded and unbounded proteins and probes are eluted during water washings which remove also buffer salts. (2) Given the hydrophobicity of C8–F resin, loaded papain, which is positioned near the resin thanks to the presence of the perfluorinated tag, finds itself during the methanol washings in an environment largely impoverished in water and can be adsorbed on the resin surface. (3) Papain is finally released from the resin only after the highly fluorophilic TFA/TFE elutions which alter the pH and ionic strength of the medium, two factors reported to be crucial in protein adsorption and desorption on and from solid surfaces.^50^

As it is known, proteins are characterized by a specific and peculiar folding, i.e., their native 3D-structures which are at the basis of their biological functions. The processes of peptide folding and unfolding, however, can be described as a complex conformational equilibrium which delineates an ensemble of accessible protein conformations which are characterized by different superficial chemophysical properties. A noteworthy example is represented by the “molten globular state”, an extended definition which includes various types of partially folded and unfolded protein states in response to local denaturing conditions. In this scenario, it can be reasonable to hypothesize that fluorous-tagged papain when immobilized onto the resin in virtue of the perfluorinated tag could also interact with the C8–F resin itself and, thanks to the discussed conformational dynamics, be physically adsorbed on it. In fact, as described in detail in the literature,^50^ the driving force for protein adsorption onto solid supports seems to be represented by the entropy gain which arises from the release of surface adsorbed water and buffer salts and from structural rearrangements inside the protein. Accordingly, after loading and water washings in which the majority of the buffer salts and unbound proteins are eluted, papain, in virtue of its binding to the fluorophilic tag, could find itself spatially near enough to the highly hydrophobic C8–F resin to allow its adsorption onto the resin beads (Figure 6). This kind of interaction would explain why the use of only methanol resulted in a not-strong-enough mobile phase to recover papain in the described experiment run with probe **1**.

Once we verified the applicability of the designed recognition/isolation method relying on the perfluorinated covalent probe **1**, the same protocol and workflow were applied this time using the fully noncovalent probe **2**. Given the low water-solubility of probe **2** and the fact that we wanted to demonstrate that a fully noncovalent approach could be applied on a sub-micromolar scale, its concentration was lowered from 4.4 mM (probe **1**) to 50 μM during the protein recognition/extraction experiments.

Obtained results are summarized in Figure 7 which shows the qualitative in-plate Bradford assay and SDS-PAGE of the collected eluates.

**Figure 7:**
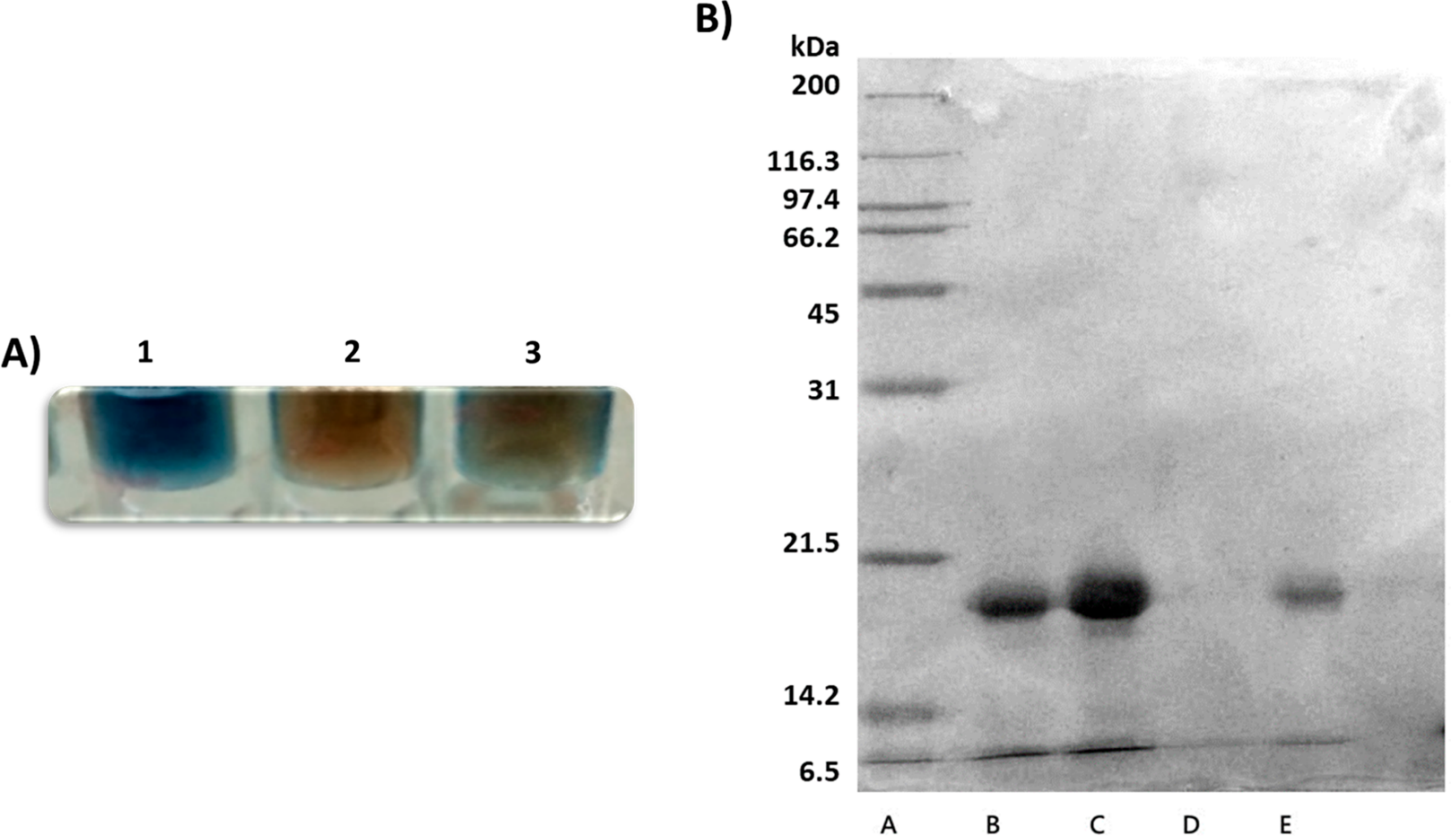
Results of the F-SPE in the presence of the noncovalent probe **2**. (A) Qualitative in-plate Bradford analysis: 1, water washings; 2, methanolic elution; 3, eluates collected in 20% TFA in TFE; (B) SDS-PAGE. A, MW markers; B, standard of papain; Fluorophilic papain isolation: C, water washings; D, methanolic eluates, E, highly fluorophilic eluates.

Given the fact that noncovalent interactions are reversible by definition and that a substoichiometric amount of probe **2** was used in this experiment, papain was successfully and selectively recovered in the highly fluorophilic eluates, although to a lower extent (*ca*. 1.5 mg) when compared to the experiment involving the covalent probe (**1**). Accordingly, the qualitative Bradford assay of the TFA/TFE eluates resulted in a less intense blue coloring (Figure 7A) as well as the reference 20 kDa band in the SDS-PAGE, which was clearly visible anyway (Figure 7B). This finding appeared to us as a logical consequence of the noncovalent interactions established between probe **2** and papain, which are by definition reversible and weaker than the ones operating in the former experiments. Moreover, since methanol is present in the medium used to load the noncovalently tagged papain to the C8–F resin, a part of the complexed probe **2** could be released from the papain active site resulting in a loss of efficacy and decrease in the amount of isolated protein material. A larger amount of unbound papain (4 mg) was in fact recovered during water washings as shown both by Bradford assay and by SDS-PAGE (Figure 7). Nevertheless, protein leakage in the methanolic elutions was not found, as eluates appeared void of any protein materials (Figure 7). This finding further supported our hypothesis of a crucial resin–protein interaction established by the presence of the fluorous tag which could act as a “linker system” able to spatially get the peptide backbone close to the resin beads resulting in its tight adsorption.

In this work, we successfully demonstrated that a convenient, fully noncovalent strategy of protein recognition and isolation can be built using PFCs as versatile and nontoxic tagging system. Using the peptide-decorated probe **2**, in fact, papain was indeed recognized by the GGYR moiety via the establishment of reversible, noncovalent interactions within its active site. Nonetheless, these reversible bonds were still able to promote the selective retention on the C8–F resin of a sufficient amount of C_7_F_15_-labeled papain to be detected in SDS-PAGE and HR-mass spectrometry after its selective release from the solid support using a strong fluorophilic eluent after water and methanolic washings. Moreover, given the fact that the molecular skeleton of our perfluorinated probes can be easily modified via synthetic chemistry, the reported strategy appears to be generally applicable for the recognition and isolation of protein targets different from papain. In fact, according to the scheme proposed in Figure 1, probe **2** has been designed to contain a perfluorinated moiety (red spheres) linked to a bioactive moiety (cyan portion) which could be easily replaced with another peptide sequence or small molecule. The nature and the specificity of the selected bioactive portion will be crucial as well for the enrichment in the desired analytes when dealing with complex biological matrices which would contain different types and concentration of contaminants. Nonetheless, our experimental workflow resulted in a quite simple and user-friendly apparatus which can be reproduced using ordinary laboratory supplies/instruments and analytical techniques.

In the light of these considerations, our investigation about noncovalent probes for protein detection and isolation will now be focused on the selective extraction of target protein from complex peptide matrices even of biological origin focusing the design of our fluoro-labeled probes on interesting and still target-less bioactive molecules.

## Supporting Information Available

Abbreviation, materials, and general information; Procedures for the F-SPE; Papain inhibition assay; Papain CD analysis; High-resolution mass spectrometry; Chemical synthesis of probe **1**; Solid-phase synthesis of probe **2** (PDF)

## Abbreviations

PFCs: perfluorinated organic compounds
F-SPE: fluorous solid phase exctration
TFE: trifluoroethanol
FTA: trifluoroacetic acid.

## References

[ref1] Chambers R. D. (2004) Fluorine in organic chemistry, pp 1–22, Chapter 1, Blackwell Publishing, Oxford.

[ref2] Smart B. E. (2001) Fluorine substituent effects (on bioactivity). J. Fluorine Chem. 109, 3–11. 10.1016/S0022-1139(01)00375-X.

[ref3] Taylor P. (2003) Ostwald ripening in emulsions: Estimation of solution thermodynamics of the disperse phase. Adv. Colloid Interface Sci. 106, 261–285. 10.1016/S0001-8686(03)00113-1. 14672850

[ref4] Parlato M. C.; Jee J. P.; Teshite M.; Mecozzi S. (2011) Synthesis, characterization, and applications of hemifluorinated dibranched amphiphiles. J. Org. Chem. 76, 6584–6591. 10.1021/jo200835y. 21736353 PMC3155948

[ref5] Tuulmets A.; Piiskop S.; Järv J.; Salmar S. (2014) Sonication effects on non-radical reactions. A sonochemistry beyond the cavitation?. Ultrason. Sonochem. 21, 997–1001. 10.1016/j.ultsonch.2013.11.001. 24279982

[ref6] Bauer J.; Rademann J. (2005) Hydrophobically assisted switching phase synthesis: the flexible combination of solid-phase and solution-phase reactions employed for oligosaccharide preparation. J. Am. Chem. Soc. 127, 7296–7297. 10.1021/ja051737x. 15898762

[ref7] Wang Y.; Dong Q.; Wang Y.; Wang H.; Li G.; Bai R. (2010) Investigation on RAFT polymerization of a y-shaped amphiphilic fluorinated monomer and anti-fog and oil-repellent properties of the polymers. Macromol. Rapid Commun. 31, 1816–1821. 10.1002/marc.201000243. 21567599

[ref8] Krafft M. P.; Riess J. G. (2009) Chemistry, physical chemistry, and uses of molecular fluorocarbon-hydrocarbon diblocks, triblocks, and related compounds. Chem. Rev. 109, 1714–1792. 10.1021/cr800260k. 19296687

[ref9] Filler R., Kobayashi Y., and Yagupolskii L. M. (1993) Organofluorine compounds in medicinal chemistry and biomedical application, Elsevier, Amsterdam.

[ref10] Yoder N. C.; Yüksel D.; Dafik L.; Kumar K. (2006) Bioorthogonal noncovalent chemistry: fluorous phases in chemical biology. Curr. Opin. Chem. Biol. 10, 576–583. 10.1016/j.cbpa.2006.10.007. 17055332

[ref11] Miller M. L.; Wesseler E. P.; Jones S. C.; Clark L. C. J. (1976) Some morphologic effects of ″inert″ particulate loading on hemopoietic elements in mice. J. Reticuloendothel. Soc. 20, 385–398. 1003403

[ref12] Ni Y.; Klein D. H.; Song D. (1996) Recent developments in pharmacokinetic modeling of perfluorocarbon emulsions. Artif. Cells, Blood Substitutes, Immobilization Biotechnol 24, 81–96. 10.3109/10731199609118876. 8907688

[ref13] Gladysz J. A., and Emnet C. (2004) Fluorous solvents and related media. In Handbook of Fluorous Chemistry (Gladysz J. A., Curran D. P., and Horvath I. T., Eds.) pp 11–23, Chapter 3, Wiley-VCH, Weinheim.

[ref14] Riess J. G. (2006) Perfluorocarbon-based oxygen delivery. Artif. Cells, Blood Substitutes, Immobilization Biotechnol 34, 567–580. 10.1080/10731190600973824. 17090429

[ref15] Waters E. A.; Chen J.; Allen J. S.; Zhang H.; Lanza G. M.; Wickline S. A. (2008) Detection and quantification of angiogenesis in experimental valve disease with integrin-targeted nanoparticles and 19-fluorine MRI/MRS. J. Cardiovasc. Magn. Reson. 10, 43 10.1186/1532-429X-10-43. 18817557 PMC2561020

[ref16] Hoerr V.; Purea A.; Faber C. (2010) NMR separation of intra- and extracellular compounds based on intermolecular coherences. Biophys. J. 99, 2336–2343. 10.1016/j.bpj.2010.06.068. 20923669 PMC3042554

[ref17] Ruiz-Cabello J.; Barnett B. P.; Bottomley P. A.; Bulte J. W. M. (2011) Fluorine (19F) MRS and MRI in biomedicine. NMR Biomed. 24, 114–129. 10.1002/nbm.1570. 20842758 PMC3051284

[ref18] Tirotta I.; Dichiarante V.; Pigliacelli C.; Cavallo G.; Terraneo G.; Bombelli F. B.; Metrangolo P.; Resnati G. (2015) 19F magnetic resonance imaging (MRI): From design of materials to clinical applications. Chem. Rev. 115, 1106–1129. 10.1021/cr500286d. 25329814

[ref19] Fox M.; Gaudet J.; Foster P. (2015) Fluorine-19 MRI contrast agents for cell tracking and lung imaging. Magn. Reson. Insights 8, 53–67. 10.4137/MRI.S23559. 27042089 PMC4807887

[ref20] Sheeran P. S.; Dayton P. A. (2012) Phase-Change contrast agents for imaging and therapy. Curr. Pharm. Des. 18, 2152 10.2174/138161212800099883. 22352770 PMC5045864

[ref21] Kiss L. E.; Kövesdi I.; Rábai J. (2001) An improved design of fluorophilic molecules: Prediction of the ln P fluorous partition coefficient, fluorophilicity, using 3D QSAR descriptors and neural networks. J. Fluorine Chem. 108, 95–109. 10.1016/S0022-1139(01)00342-6.

[ref22] Sadtler V. M.; Giulieri F.; Krafft M. P.; Riess J. G. (1998) Micellization and adsorption of fluorinated amphiphiles: questioning the 1CF_2_≈1.5 CH_2_ rule. Chem. - Eur. J. 4, 1952–1956. 10.1002/(SICI)1521-3765(19981002)4:10<1952::AID-CHEM1952>3.0.CO;2-V.

[ref23] Krafft M. P. (2001) Fluorocarbons and fluorinated amphiphiles in drug delivery and biomedical research. Adv. Drug Delivery Rev. 47, 209–228. 10.1016/S0169-409X(01)00107-7. 11311993

[ref24] Kirsch P. (2004) Modern fluoroorganic chemistry. Synthesis, reactivity, applications, pp 1–23, Chapter 1, Wiley-VCH, Weinheim.

[ref25] Percec V.; Imam M. R.; Bera T. K.; Balagurusamy V. S. K.; Peterca M.; Heiney P. A. (2005) Self-assembly of semifluorinated Janus-dendritic benzamides into bilayered pyramidal columns. Angew. Chem., Int. Ed. 44, 4739–4745. 10.1002/anie.200501254. 15995996

[ref26] Maeda H.; Matsumura Y. (1986) A new concept for macromolecular therapeutics in cancer chemotherapy: mechanism of tumoritropic accumulation of proteins and the antitumor agent smancs. Cancer Res. 46, 6387–6392. 2946403

[ref27] Ping M.; Russel J. M. (2013) Paclitaxel nano-delivery systems: a comprehensive review. J. Nanomed. Nanotechnol. 4, 1–35. 10.4172/2157-7439.1000164. PMC3806207 24163786

[ref28] Frank D.; Tyag i C.; Tomar L.; Choonara Y. E.; du Toit L. C.; Kumar P.; Penny C.; Pillay V. (2014) Overview of the role of nanotechnological innovations in the detection and treatment of solid tumors. Int. J. Nanomed. 9, 589–613. 10.2147/IJN.S50941. PMC3904834 24489467

[ref29] Allen T. M.; Cullis P. R. (2004) Drug delivery systems: entering the mainstream. Science 303, 1818–1822. 10.1126/science.1095833. 15031496

[ref30] Stähler K.; Selb J.; Candau F. (1999) Multicompartment polymeric micelles based on hydrocarbon and fluorocarbon polymerizable surfactants. Langmuir 15, 7565–7576. 10.1021/la990431z.

[ref31] Zhang W.; Curran P. D. (2006) Synthetic applications of fluorous solid-phase extraction (F-SPE). Tetrahedron 62, 11837–11865. 10.1016/j.tet.2006.08.051. 18509513 PMC2396515

[ref32] Dandapani S. (2006) Recent applications of fluorous separation methods in organic and bioorganic chemistry. QSAR Comb. Sci. 25, 681–688. 10.1002/qsar.200640051.

[ref33] Curran D. P. (2008) Fluorous tags unstick messy chemical. Science 321, 1645–1646. 10.1126/science.1158721. 18801990

[ref34] Hayama T.; Yoshida H.; Yamaguchi M.; Nohta H. (2014) Fluorous affinity-based separation techniques for the analysis of biogenic and related molecules. J. Pharm. Biomed. Anal. 101, 151–160. 10.1016/j.jpba.2014.04.035. 24865313

[ref35] Brittain S. M.; Ficarro S. B.; Brock A.; Peters E. C. (2005) Enrichment and analysis of peptide subsets using fluorous affinity tags and mass spectrometry. Nat. Biotechnol. 23, 463–468. 10.1038/nbt1076. 15768030

[ref36] Guerrera I. C.; Kleiner O. (2005) Application of mass spectrometry in proteomics. Biosci. Rep. 25, 71–93. 10.1007/s10540-005-2849-x. 16222421

[ref37] Go E. P.; Uritboonthai W.; Apon J. V.; Trauger S. A.; Nordstrom A.; O’Maille G.; Brittain S. M.; Peters E. C.; Siuzdak G. (2007) Selective metabolite and peptide capture/mass detection using fluorous affinity tags. J. Proteome Res. 6, 1492–1499. 10.1021/pr060608s. 17343404 PMC2530906

[ref38] Ying W.; Perlman D. H.; Li L.; Théberge R.; Costello C. E.; McComb M. E. (2009) Highly efficient and selective enrichment of peptide subsets combining fluorous chemistry with reversed-phase chromatography. Rapid Commun. Mass Spectrom. 23, 4019–4030. 10.1002/rcm.4343. 19924777 PMC3584324

[ref39] Flaxman H. A.; Woo C. M. (2018) Mapping the small molecule interactome by mass spectrometry. Biochemistry 57, 186–193. 10.1021/acs.biochem.7b01038. 29083874

[ref40] Ko K. S.; Jaipuri F. A.; Pohl N. L. (2005) Fluorous-based carbohydrate microarrays. J. Am. Chem. Soc. 127, 13162–13163. 10.1021/ja054811k. 16173741

[ref41] Babu Kumar A.; Anderson J. M.; Manetsch R. (2011) Design, synthesis and photoactivation studies of fluorous photolabels. Org. Biomol. Chem. 9, 6284–6292. 10.1039/c1ob05748k. 21785789

[ref42] Claesener M.; Breyholz H. J.; Hermann S.; Faust A.; Wagner S.; Schober O.; Schäfers M.; Kopka K. (2012) Efficient synthesis of a fluorine-18 labeled biotin derivative. Nucl. Med. Biol. 39, 1189–1194. 10.1016/j.nucmedbio.2012.08.001. 22998841

[ref43] Zhao M.; Deng C. (2016) Designed synthesis of fluorous-functionalized magnetic mesoporous microspheres for specific enrichment of phosphopeptides with fluorous derivatization. Proteomics 16, 1051–1058. 10.1002/pmic.201500323. 26800430

[ref44] Saito S.; Murai Y.; Usuki S.; Yoshida M.; Hammam M. A. S.; Mitsutake S.; Yuyama K.; Igarashi Y.; Monde K. (2017) Synthesis of nontoxic fluorous sphingolipids as molecular probes of exogenous metabolic studies for rapid enrichment by fluorous solid phase extraction. Eur. J. Org. Chem. 2017, 1045–1051. 10.1002/ejoc.201601302.

[ref45] Elshani S.; Kobzar E.; Bartsch R. A. (2000) Macrocyclic ligands with partially fluorinated sidearms: Synthesis and metal ion complexation. Tetrahedron 56, 3291–3301. 10.1016/S0040-4020(00)00248-9.

[ref46] LaLonde J. M.; Zhao B.; Smith W. W.; Janson C. A.; DesJarlais R. L.; Tomaszek T. A.; Carr T. J.; Thompson S. K.; Oh H. J.; Yamashita D. S.; Veber D. F.; Abdel-Meguid S. S. (1998) Use of papain as a model for the structure-based design of cathepsin K inhibitors: Crystal structures of two papain-inhibitor complexes demonstrate binding to S’-subsites. J. Med. Chem. 41, 4567–4576. 10.1021/jm980249f. 9804696

[ref47] Baines B. S.; Brocklehurst K. (1979) A necessary modification to the preparation of papain from any high-quality latex of Carica papaya and evidence for the structural integrity of the enzyme produced by traditional methods. Biochem. J. 177, 541–548. 10.1042/bj1770541. 435250 PMC1186404

[ref48] Baines B. S.; Brocklehurst K. (1978) A spectrophotometric method for the detection of contaminant chymopapains in preparations of papain. Selective modification of one type of thiol group in the chymopapains by a two-protonic-state reagent. Biochem. J. 173, 345–347. 10.1042/bj1730345. 687376 PMC1185781

[ref49] Funk M. O.; Nakagawa Y.; Skochdopole J.; Kaiser E. T. (1979) Affinity chromatographic purification of papain. A reinvestigation. Int. J. Pept. Protein Res. 13, 296–303. 10.1111/j.1399-3011.1979.tb01883.x. 429102

[ref50] Rabe M.; Verdes D.; Seeger D. (2011) Understanding protein adsorption phenomena at solid surfaces. Adv. Colloid Interface Sci. 162, 87–106. 10.1016/j.cis.2010.12.007. 21295764

