# Supplementary material for: Perfluorinated Probes for Noncovalent Protein Recognition and Isolation
Source: Bioconjug Chem. 2020 Jan 11;31(3):513–9. doi: 10.1021/acs.bioconjchem.9b00846 (PMC7993633; doi:10.1021/acs.bioconjchem.9b00846)
Supplement: Supplementary file 1 — bc9b00846_si_001.pdf [file bc9b00846_si_001.pdf]

Supplementary Information for

Perfluorinated probes for *non-covalent* protein recognition and isolation

Ivan Bassanini,[†] Corinna Galli,[†] Erica E. Ferrandi,[‡] Fabiana Vallone,[§] Annapaola Andolfo,[§] Sergio Romeo[†,*]

† Dipartimento di Scienze Farmaceutiche, Università degli Studi di Milano, Via Mangiagalli 25 20133-Milano, IT; ‡ Istituto di Chimica del Riconoscimento Molecolare - Consiglio Nazionale delle Ricerche, Via Mario Bianco 9 20131-Milano, IT; [§] ProMiFa, Protein Microsequencing Facility, Ospedale San Raffaele, Via Olgettina 60 20132-Milano, IT

INDEX

1. *Abbreviations & General Information* 2
2. *CD analysis* 3
3. *General procedure for the F-SPE of inhibited papain* 4
4. *Papain inhibition assay* 5
5. *High-resolution mass spectrometry* 6
6. *Synthetic procedures* 9
7. *Solid Phase Synthesis: general methods* 11

1. Abbreviations & General Information

THF: tetrahydrofuran;
DCM: dichloromethane;
DMF: dimethylformamide;
TBTU: 2-(1H-benzotriazole-1-yl)-1,1,3,3-tetramethylaminium tetrafluoroborate;
HOBt: 1-hydroxybenzotriazole hydrate;
NMM: *N*-methyl morpholine;
Boc: tert-butyloxycarbonyl
r.t.: room temperature
iBuOCOCl: isobutyl chloroformate
NMP: *N*-methyl-2-pyrrolidone
Fmoc: fluorenylmethyloxycarbonyl

All the reagents and solvents were purchased from Sigma-Aldrich® and BioRad® and used without any further purification. TLC was carried out on Merck precoated 60 F254 plates using UV light and dipping with ethanol/phosphomolybdic acid 10%. Flash column chromatography was performed using silica gel 60 (0.040-0.063 mm, Merck).
The resin used for the fluorophilic solid-phase extraction, a silica gel C8-reversed phase, was purchased by Sigma-Aldrich®, catalogue number: 18387-10G-F.
Organic phases were dried over anhydrous sodium sulphate.
Concentrations were performed under diminished pressure (1-2 kPa) at a bath temperature of 40 °C.
Purities of final compounds were determined by HPLC using $CH_3CN/H_2O + CF_3COOH$ gradient and a Purospher RP18 5 µm column on a Hitachi Elite Lachrom Instrument equipped with a DAD detector.
Inhibition kinetics and were recorder at 20 °C on a Jasco V-530 UV/VIS spectrophotometer (Easton, MD, USA);

Papain elution was monitored by SDS-PAGE analysis (15%T, 2.6% C) according to the method of Laemmli. The molecular weight protein standard mixture from Bio-Rad (Karlsruhe, Germany) was used as reference. Gels were stained for protein detection with Coomassie Brilliant Blue.

2. CD analysis

Parameters
- Temperature: r.t.
- Concentration: 0.075 mg mL^{-1}
- Solvent: PB buffer 85 mM pH 6 or 25, 50 and 90% v/v of MeOH in the mentioned buffer
- Sensitivity: 100 mdeg
- λ: from 250 nm to 190 nm
- Data pick: 0.1 nm
- Scanning mode: continuous;
- Scanning speed: 20 nm/min
- Response: 1 sec

- Bond width: 1
- Accumulation: 3

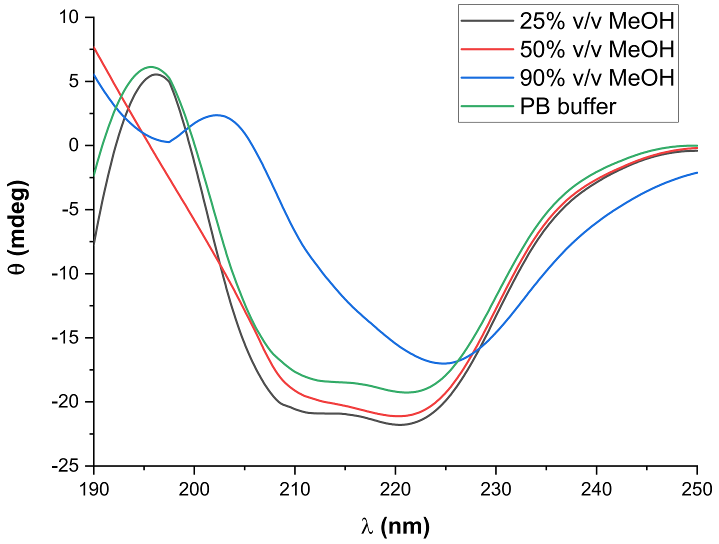

Figure S1. CD-analysis conducted of a 0.075 mg mL^{-1} solution of papain conducted in the presence of 25%, 50% and 90% v/v amount of methanol.

Papain showed its native folding (*i.e.* an α-helix, *green* line) when dissolved in PB buffer while a complete unfolding was found in the extreme denaturing media represented by a 90% v/v mixture of methanol and PB buffer (*blue* line). Most importantly to us, papain was found to be completely folded in the presence of the 25% v/v of methanol (*black* line) and still partially folded when dissolved in a 50% v/v mixture of methanol and PB buffer (*red* line).

3. General procedure for the F-SPE of inhibited papain

Papain inhibition

The proper amount of a 56 mM DMSO solution of **probe 1** (final concentration = 5 mM) or of a 5 mM DMSO solution of **probe 2** (final concentration = 50 μM) was added to a solution of activated papain (*i.e.* incubation in a thermoshaker at 25 °C for 45 min) composed as follows: papain (145 μM), β-mercaptoethanol (21 mM) and EDTA (9 mM) dissolved in PB-buffer (85 mM, pH 6). The obtained mixture was incubated in a thermoshaker (25 °C, 190 rpm) for 15 minutes

Loading and fluorophilic extraction

The solution of inhibited papain was at first diluted (1.25x) with a 1:1 mixture of water and methanol ([methanol] = 25% v/v) and then loaded into a plastic column filled with the C8 RP-perfluorinated resin (1 g_{resin}/4.8mg_{papain}) to be incubated again for 5 minutes in a thermoshaker (25 °C, 150 rpm).
After incubation and loading, elutions and fractioning was made as follows (CV = column volume):

- **A:** 0.5 CV with water
- **B:** 4 CV with water
- **C:** 4 CV with methanol
- **D:** 4 CV with a 20% v/v TFA solution in TFE
- **E:** 6 CV with acetone

The collected fractions, when necessary were concentrated *in vacuo* removing all the organic solvents, taken up with water (*ca* 1 mL) and analyzed qualitatively by means of a in-plate colorimetric Bradford® assay to detect the presence of eluted proteins (*i.e.* papain) using a solution of activated papain, the activating buffer and DAMK-1 or GGTA-1 as references. Papain-containing samples were then lyophilized and analyzed by means of SDS-PAGE on acrylamide gel and/or HR-mass spectrometry.

4. Papain inhibition assay

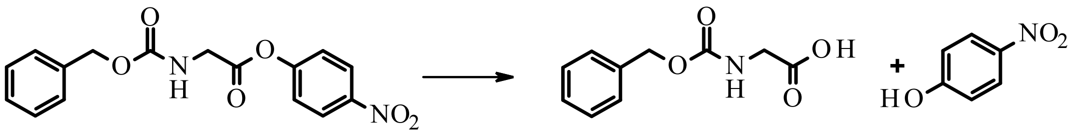

Scheme S1. 4-nitrophenyl 2-(((benzyloxy)carbonyl)amino)acetate hydrolysis.

Reagents and condition for ***Probe 1***

Reference: papain (145 µM), β-mercaptoethanol (21 mM) and EDTA (9 mM) dissolved in PB-buffer (85 mM, pH 6) + 4-nitrophenyl 2-(((benzyloxy)carbonyl)amino)acetate (4.4 mM);

Sample: papain (145 µM), β-mercaptoethanol (21 mM) and EDTA (9 mM) dissolved in PB-buffer (85 mM, pH 6) + 4-nitrophenyl 2-(((benzyloxy)carbonyl)amino)acetate (4.4 mM) + **probe 1** (4.4 mM); λ = 340 nm;

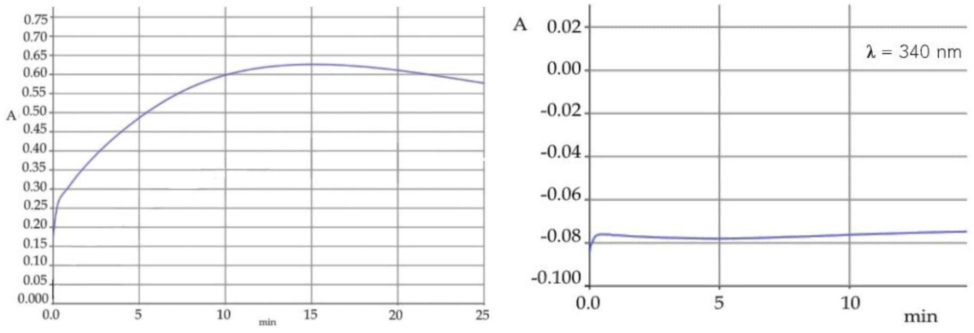

Figure S2. (*left*) native hydrolytic activity of papain; (*right*) papain inhibition in the presence of **probe 1**.

Reagents and condition for ***Probe 2***

Reference: papain (50 µM), β-mercaptoethanol (21 mM) and EDTA (9 mM) dissolved in PB-buffer (85 mM, pH 6) + 4-nitrophenyl 2-(((benzyloxy)carbonyl)amino)acetate (1.5 mM);

Sample: papain (50 µM), β-mercaptoethanol (21 mM) and EDTA (9 mM) dissolved in PB-buffer (85 mM, pH 6) + 4-nitrophenyl 2-(((benzyloxy)carbonyl)amino)acetate (1.5 mM) + **probe 2** (50 and 150 µM); λ = 340 nm;

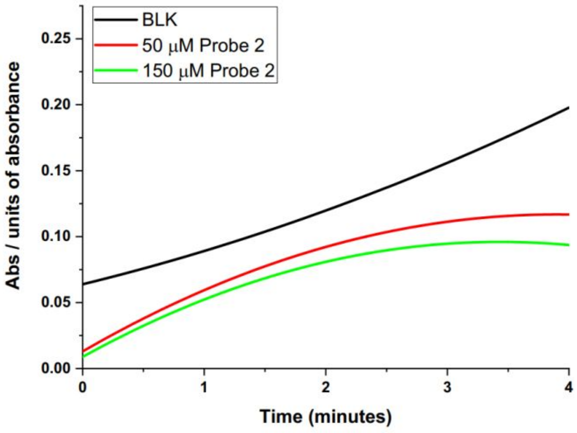

Figure S3. Native hydrolytic activity of papain (back line); papain inhibition in the presence of 50 µM (red line) and 150 µM (green line) **probe 2**.

5. High-resolution mass spectrometry

Papain-containing lyophilized samples from step 2 were sequentially reduced, alkylated and digested overnight with sequencing-grade trypsin (1:50, w/w). Aliquots of the sample containing tryptic peptides were desalted using StageTip C18 (Thermo Scientific) and analysed by nLC-MS/MS using a Q-Exactive mass spectrometer (Thermo Scientific, Bremen, Germany) equipped with a nano-electrospray ion source (Proxeon Biosystems) and a nUPLC Easy nLC 1000 (Proxeon Biosystems). Peptide separations occurred on a homemade (75 µm i.d., 12 cm long) reverse phase silica capillary column, packed with 1.9 µm ReproSil-Pur 120 C18-AQ (Dr. Maisch GmbH, Germany). A gradient of eluents A (water with 0.1% v/v formic acid) and B (acetonitrile with 0.1% v/v formic acid) was used to achieve separation (300 nL/min flow rate), from 0% B to 45% B in 45 minutes. Full scan spectra were acquired with the lock-mass option, resolution set to 70,000 and mass range from *m*/*z* 300 to 2000 Da. The ten most intense doubly and triply charged ions were selected and fragmented. All MS/MS samples were analysed using Mascot search engine (version 2.6, Matrix Science) to search the SwissProt Complete Proteome_2016_10 (552,884 sequences; 197,760,918 residues), taxonomy Viridiplantae. Searches were performed with the following settings: trypsin as proteolytic enzyme; 3-missed cleavages allowed; addition of 677.16 Da on cysteine, due to the addition of the fluorinated papain inhibitor LC-12, as fixed modification; protein N-terminus-acetylation, methionine oxidation and carbamidomethylation on cysteine as variable modifications; mass tolerance was set to 5 ppm and to 0.02 Da for precursor and fragment ions, respectively.

Red-CAM, in-solution digestion: Trypsin

Protein View: PAPA3_CARPA
Caricain OS=Carica papaya PE=1 SV=2
Protein sequence coverage: 34%, (with the respect of the overall sequence)
Score: 1381

Mature Protein 133-348

1	MAMIPSISKL	LFVAICLFVH	MSVSFGDFSI	VGYSQDDLTS	TERLIQLFNS
51	WMLNHNKFYE	NVDEKLYRFE	IFKDNLNYID	ETNKKNNSYW	LGLNEFADLS
101	NDEFNEKYVG	SLIDATIEQS	YDEEFINEDT	VNLPENVDWR	K**KGAVTPVR**H
151	QGSCGSCWAF	SAVATVEGIN	KIRTGK**LVEL**	**SEQELVDCER**	RSHGCK**GGYP**
201	**PYALEYVAK**N	GIHLRSKYPY	KAKQGTCRAK	**QVGGPIVK**TS	GVGR**VQPNNE**
251	**GNLLNAIAKQ**	**PVSVVVESKG**	**RPFQLYK**GGI	FEGPCGTK**VD**	**HAVTAVGYGK**
301	SGGKGYILIK	**NSWGTAWGEK**	GYIRIK**RAPG**	**NSPGVCGLYK**	**SSYYPTKN**

Figure S4. MS/MS-based identification of “papain protease 3”.

Red-CAM, in-solution digestion: Trypsin

Protein View: PAPA4_CARPA
Papaya proteinase 4 OS=Carica papaya PE=1 SV=3
Protein sequence coverage: 32% , (with the respect of the overall sequence)
Score: 719

Mature Protein 133-348

```
  1 MAIICSFSKL LFVAICLFGH MSLSYCDFSI VGYSQDDLTS TERLIQLFNS
 51 WMLKHNKNYK NVDEKLYRFE IFKDNLKYID ERNKMINGYW LGLNEFSDLS
101 NDEFKEKYVG SLPEDYTNQP YDEEFVNEDI VDLPESVDWR AKGAVTPVKH
151 QGYCESCWAF STVATVEGIN KIKTGNLVEL SEQELVDCDK QSYGCNRGYQ
201 STSLQYVAQN GIHLRAKYPY IAKQQTCRAN QVGGPKVKTN GVGRVQSNNE
251 GSLLNAIAHQ PVSVVVESAG RDFQNYKGGI FEGSCGTKVD HAVTAVGYGK
301 SGGKGYILIK NSWGPGWGEN GYIRIRRASG NSPGVCGVYR SSYYPIKN
```

Figure S5. MS/MS-based identification of "papain protease 4"

Red-CAM, in-solution digestion: Trypsin

Protein View: PAPA2_CARPA
Chymopapain OS=Carica papaya PE=1 SV=2
Protein sequence coverage: 27%, (with the respect of the overall sequence)
Score: 477

Mature Protein 135-352

```
  1 MATMSSISKI IFLATCLIIH MGLSSADFYT VGYSQDDLTS IERLIQLFDS
 51 WMLKHNKIYE SIDEKIYRFE IFRDNLMYID ETNKKNNSYW LGLNGFADLS
101 NDEFKKKYVG FVAEDFTGLE HFDNEDFTYK HVTNYPQSID WRAKGAVTPV
151 KNQGACGSCW AFSTIATVEG INKIVTGNLL ELSEQELVDC DKHSYGCKGG
201 YQTTSLQYVA NNGVHTSKVY PYQAKQYKCR ATDKPGPKVK ITGYKRVPSN
251 CETSFLGALA NQPLSVLVEA GGKPFQLYKS GVFDGPCGTK LDHAVTAVGY
301 GTSDGKNYII IKNSWGPNWG EKGYMRLKRQ SGNSQGTCGV YKSSYYPFKG
351 FA
```

Figure S6. MS/MS-based identification of "papain protease 2"

Red-CAM, in-solution digestion: Trypsin

Protein View: PAPA1_CARPA
Papain OS=Carica papaya PE=1 SV=1
Protein sequence coverage: 22%, (with the respect of the overall sequence)
Score: 225

Mature Proteina 134-345

```
  1  MAMIPSISKL  LFVAICLFVY  MGLSFGDFSI  VGYSQNDLTS  TERLIQLFES
 51  WMLKHNKIYK  NIDEKIYRFE  IFKDNLKYID  ETNKKNNSYW  LGLNVFADMS
101  NDEFKEKYTG  SIAGNYTTTE  LSYEEVLNDG  DVNIPEYVDW  RQKGAVTPVK
151  NQGSCGSCWA  FSAVVTIEGI  IKIRTGNLNE  YSEQELLDCD  RRSYGCNGGY
201  PWSALQLVAQ  YGIHYRNTYP  YEGVQRYCRS  REKGPYAAKT  DGVRQVQPYN
251  EGALLYSIAN  QPVSVVLEAA  GKDFQLYRGG  IFVGPCGNKV  DHAVAAVGYG
301  PNYILIKNSW  GTGWGENGYI  RIKRGTGNSY  GVCGLYTSSF  YPVKN
```

Figure S7. MS/MS-based identification of “papain protease 1”

6. Synthetic procedures

a_1) *Peptide coupling: HOBt chemistry*

A solution of the desired primary amine (1.0 equiv., 500 mM) and the selected *N*-Boc aminoacid (1.1 equiv.) was prepared in a proper solvent (THF, DCM or DMF) and cooled to 0 °C. TBTU (1.1 equiv) and HOBt (1.5 equiv.) were then added and the pH set at 8 using NMM. The reaction was magnetically stirred at room temperature overnight. After that, the solvent was removed in *vacuo* and the residue was taken up with DCM (5mL $mmol^{-1}{}_{amine}$). The obtained solution was then washed three times with a saturated aqueous solution of $NaHCO_3$, two times with water and brine, dried over Na_2SO_4 and finally concentrated again in *vacuo*. If necessary, the crude product was purified by flash column chromatography on silica gel suing a gradient of MeOH in DCM as mobile phase or it was used without any further purifications.

a_2) *Peptide coupling: DIC chemistry*

DIC (1.5 equiv.) and DMPA (1/20 equiv.) were added to an ice-cooled solution of the desired primary amine (1.0 equiv., 50 mM) and the selected perfluorinated acid (1.4 equiv.) prepared in dry DCM. The pH set at 8 using NMM and the reaction was magnetically stirred at room temperature overnight. After that, the solvent was removed in *vacuo* and the residue was taken up with DCM (5mL $mmol^{-1}{}_{amine}$). The obtained solution was then washed two times with water and brine, dried over Na_2SO_4 and finally concentrated again in *vacuo*. The
crude product was purified by silica gel column chromatography (gradient of MeOH in DCM).

b) *Deprotection of N-Boc amines*

The *N*-Boc amine was dissolved in a 4 N dioxane solution of HCl (10mL $g^{-1}{}_{amine}$) and stirred at room temperature for 3 hours. After that, the solvent was removed by in *vacuo* concentration affording the corresponding primary amine which was used without any further purifications.

c) *Hydrogenolysis*

Benzyl-protected compound was dissolved in methanol (10 mL $mmol^{-1}$) and then 10% Pd/C (80 mg $mmol^{-1}$) was added. The mixture was reacted under hydrogen at r.t. and atmospheric pressure for 1 day. The mixture was filtered and the solvent removed under reduced pressure. The deprotected product is used in the following step without further purifications.

d) *Alkylation*

Under argon atmosphere at 0°C, a THF solution of a primary alcohol (0.5 equiv., 200 mM) was added dropwise to a suspension of NaH (3 equiv.) prepared in dry THF (30 mL g^{-1}) and the resulting mixture was magnetically stirred for 1 h at room temperature. After that, a THF solution (400 mM) bromoacetic (1 equiv.) was dropped into the reacting mixture which was stirred for 24 h. The solvent was then removed *in vacuo*, 2M HCl was added and the desired product was extracted with DCM (three times). The combined organic layers were finally dried over Na_2SO_4 and concentrated again *in vacuo* affording the desired product which was used without any further purifications.

e) *p-Tolylsulfonylmethylnitrosamine (Diazald®) synthesis*

A total of 100 g (0.53 mmol) of *p*-toluenesulfonyl chloride was divided into three portions of 59.4 g, 28.1 g and 12.5 g. The first portion was added during about 5 minutes to 52.8 mL (0.63 mmol) of 40% aqueous methylamine contained in a round-bottomed flask. The mixture was allowed to heat to 80-90 °C in order to maintain the sulfonylmethyl amide in a molten condition. As soon as the mixture became acidic, 15.6 mL of 50% aqueous NaOH (23.5 g NaOH dissolved in 47 ml of H_2O) was added carefully. This was followed immediately by gradual addition of the second portion of the *p*-toluenesulfonyl chloride as before. When the mixture became acidic again, 7.8 mL of the aqueous NaOH solution was added, followed by the final portion of *p*-toluenesulfonyl chloride. After the mixture has again become acidic, the remainder of the aqueous NaOH solution was added. The liquid phase of the final mixture should be alkaline, otherwise methylamine was added to render the mixture basic. The mixture was stirred vigorously for 15 minutes. The hot reaction mixture was poured into 470 mL of glacial acetic acid and the flask was rinsed clean with 80 mL of acetic acid. The solution was cooled in an ice bath to about 5 °C and an aqueous solution of $NaNO_2$ (39.1 g dissolved in 78 mL of H2O) is added from a dropping funnel during about 45 minutes. The temperature of the mixture must be kept below 10 °C, and stirring was continued for 15 minutes after addition was completed. During the reaction, the nitroso compound separated as a yellow crystalline product. 315 mL of H_2O was added to the mixture. Then, the precipitate was separated by filtration, pressed on the funnel, and washed with about 155 mL of H_2O. The product was transferred to a beaker, stirred well with about 125 mL of H_2O, then filtered and washed again on the funnel. After drying to constant weight under *vacuo*, the product melts in the range between 55 and 60 °C.

f) *Diazomethane synthesis*

In a round-bottom flask equipped with graduated dropping funnel and Claisen condenser, KOH aqueous solution (3 g dissolved in 5 mL of water), EtOH 95% (5 mL) and Et_2O (5 mL) was added and stirred vigorously. The mixture was heated to 70 °C and, when Et_2O started to evaporate, am ether solution of Diazald® (11.25 g in 65 mL of Et_2O) was added dropwise. The evaporating solvent was then condensed and collected in a round-bottom flask kept at -20 °C. Diazomethane was formed and collected as diethyl ether solution in the round-bottom flask at -20 °C. At the end of the

reaction, the ether solution of diazomethane (~ 50 mL) was ready to be used in the following step without further purifications. All glassware was neutralized with acetic acid.

g) *Diazomethyl ketone synthesis*
*i*BuOCOCl (1.1 equiv.) was added to a cooled (-15 °C) solution of carboxylic acid (1.0 equiv.) and NMM (1.1 equiv.) in dry THF (4.7 mL $mmol^{-1}{}_{acid}$). The reaction was then stirred for 15 minutes at r.t. The mixture was filtered, the liquid was cooled to -70 °C and the diethyl ether solution of diazomethane was finally added. The mixture was allowed to cool at r.t. and reacted overnight. The solvent was evaporated under reduced pressure and the residue taken-up in Et_2O. The organic layer was then washed once with a saturated aqueous solution of $NaHCO_3$ and once with brine. The organic phase was dried over Na_2SO_4 and evaporated under reduced pressure.

7. Solid Phase Synthesis: general methods

Materials

Resin: 2-chlorotrityl chloride, loading 1.6 mmol g^{-1}

N-Fmoc protected aminoacids

Fmoc-cleavage cocktail (a): 20% solution of piperidine in dry DMF (ex: 10 mL of piperidine dissolved in 40 mL of dry DMF, stored at +4 °C).

Coupling cocktail (b): A 450 mM solution of TBTU (PM: 321.1, 5.78 g)/HOBt (PM: 135.1, 2.75 g) solution was prepared in dry DMF (40 mL, stored at +4 °C).

Peptide cleavage cocktail (c): Solution of 84% TFA, 5% distilled water, 5% phenol, 5% thioanisole, 1% TIS (Triisopropylsilane)

Coupling cocktail (d): DMF (7.5 mL, dry) solution of DIC (LABR387) (450 mM, 4.5 mmol, PM: 126.20, d: 0.81 g/mL, 0.7 mL) and DIMAP (FR171) (22.5 mM, PM:, 29 mg), stored at +4 °C.

General Procedures

h) *First coupling. N*-Fmoc-AA-OH (1.2 equiv. with respect to the resin) and DIPEA (4 eq with respect to the acid) were dissolved in dry DCM (10 $mL^{-1}{}_{resin}$). This solution was added to the swelled-resin which was agitated for 120 min. After that, the resin was washed with 3x DCM/MeOH/DIPEA (17:2:1), 3x DCM, 2x DMF, 2x DCM and dried in *vacuo* overnight over KOH.

i) *Kiser test.* Some grains of dried resin were placed in a transparent vessel to which '3 drops' of the following solutions are added:

- 80% ethanolic phenol;
- 5% ethanolic ninhydrin;
- KCN_{acq} in pyridine (20 μM)

The vessel was placed for 5 min at 100 °C. If the grains turned blue free amine groups were still present, if transparent all of them were reacted.

j) *Coupling reaction***:** The Fmoc-amino acid loaded resin was incubated with the *Fmoc-cleavage cocktail (a)* for 5 minutes (10 mL $g^{-1}{}_{resin}$). After drying in *vacuo*, the resin was washed 6x with DMF and 2x with EtOH and a Kiser test was run. If positive, the desired of *N*-Fmoc-protected aminoacid (3 equiv.) was dissolved in 2.9 mL of *coupling cocktail (b)* (4 equiv., 1.31 mmol of TBTU/HOBt) and 461 μL of DIPEA (8 eq, equiv.) was added to the resulting solution and the obtained mixture was incubated and shacked with the resin for 55 minutes. After washing (under shacking) with 6x with DMF and drying with 2x EtOH a Kiser test was run.

k) *Coupling reaction with DIC-chemistry:* The Fmoc-amino acid loaded resin was incubated with the *Fmoc-cleavage cocktail (a)* for 5 minutes (10 mL $g^{-1}{}_{resin}$). After drying in *vacuo*, the resin was washed 6x with DMF and 2x with EtOH and a Kiser test was run. If positive, $CF_3(CF_2)_2$-CH_2OCH_2-COOH (1.4 equiv, 0.46 mmol) was dissolved in 1 mL of *coupling cocktail (d)* and 148 μL of NMM (4 equiv) were added to the resulting solution. The obtained mixture was incubated and shacked with the resin for 120 minutes. After washing (under shacking) with 6x with DMF and drying with 2x EtOH a Kiser test was run.

l) *Peptide cleavage: peptide cleavage cocktail (c)* was added to the resin (15 mL/g resin) which was shacked for 3 hours. The resin was removed by filtration and washed 2x with TFA. A 75:25 mixture of cold TMBE and petroleum ether (from *ca* 10x dilution) was added dropwise to the combined filtrates to trigger peptide precipitation. Sometimes, TFA concentration *in vacuo* further cooling in ice was needed to ensure precipitation.

- **Probe 1: synthesis**

2-((2,2,3,3,4,4,5,5,6,6,7,7,8,8,8-pentadecafluorooctyl)oxy)acetic acid (A)

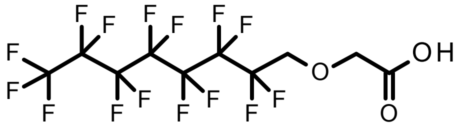

Perfluorinated acid **A** (723.8 mg, 1.53 mmol, white solid**)** was prepared from bromoacetic acid (486.3 mg, 3.5 mmol) and commercially available 2,2,3,3,4,4,5,5,6,6,7,7,8,8,8-pentadecafluorooctan-1-ol following (700.0 mg, 1.7 mmol) general procedure **D**.
^{1}**H NMR** (300 MHz, Chloroform-*d*), δ: 4.33 (s, 2H), 4.13 (t, J = 13.6 Hz, 2H); ^{19}**F NMR** (282 MHz, Chloroform-*d*), δ: -80.78 (t, J = 9.9 Hz, 3F); -119.68, -120.07 (m, 2F); -121.85, -122.42 (m, 4F); -122.64, -123.09 (m, 2F); -123.20, -123.49 (m, 2F); -125.96, -126.34 (m, 2F); **ESI-MS**, *m/z*: 457.00 $[M-H]^-$.

Benzyl (tert-butoxycarbonyl)-L-phenylalanylglycinate (i)

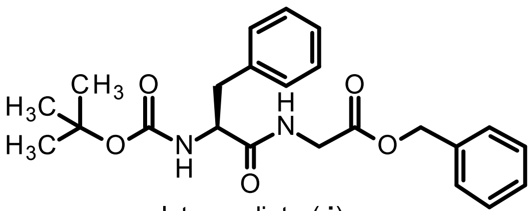

Intermediate (**i**)

According to general coupling procedure $\mathbf{a_1}$, Boc-L-Phe-OH (2.59 g, 9.78 mmol) was reacted with Gly benzyl ester *p*-toluenesulfonate salt (3.00 g, 8.89 mmol). Intermediate **i** was obtained (2.38 g, 7.95 mmol, 89%, white solid) after a flash column chromatography on silica gel using a gradient of MeOH in DCM (R*f* = 0.81, DCM/MeOH 9:1).
^{1}**H NMR** (300 MHz, Chloroform-*d*), δ: 7.45 – 7.14 (m, 10H), 6.41 (t, J = 5.4 Hz, 1H), 5.16 (s, 2H), 4.96 (bs, 1H), 4.40 (t, J = 7.4 Hz, 1H), 4.08 (dd, J = 18.3, 5.4 Hz, 1H), 3.96 (dd, J = 18.4, 5.1 Hz, 1H), 3.09 (d, J = 7.5 Hz, 2H), 1.39 (s, 9H); **ESI-MS**, *m/z*: 411.22 $[M-H]^-$.

Benzyl (tert-butoxycarbonyl)-L-leucyl-L-phenylalanylglycinate (ii)

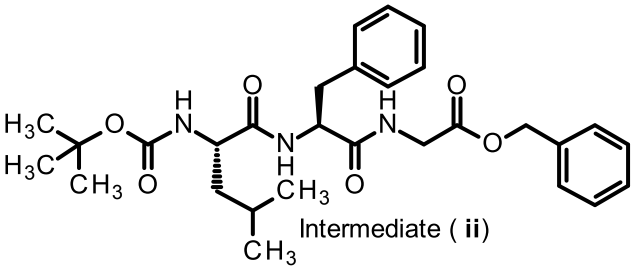

Intermediate (**ii**)

Intermediate (**1**) (2.38 g, 7.95 mmol) was converted into the corresponding primary amine in quantitative yield following general procedure **b**. Deprotected (**1**) was reacted with Boc-L-Leu Gly (2.18 g, 8.76 mmol) according to general procedure $\mathbf{a_1}$. Intermediate **ii** was obtained (2.78 g, 5.29 mmol, 67%, white solid) after a flash column chromatography on silica gel using a gradient of MeOH in DCM (R*f* = 0.88, DCM/MeOH 9:1).
^{1}**H NMR** (300 MHz, Chloroform-*d*), δ: 7.43 – 7.16 (m, 11H), 6.68 (bs, 1H), 6.48 (d, *J* = 8.1 Hz, 1H), 5.15 (s, 2H), 4.79 – 4.62 (m, 2H), 4.08 (dd, *J* = 18.0, 6.0 Hz, 1H), 3.91 (dd, *J* = 18.0, 6.0 Hz, 1H), 3.14 (d, *J* = 4.7 Hz, 2H), 1.68 – 1.48 (m, 3H), 1.38 (s, 9H), 0.89 (t, *J* = 6.4 Hz, 6H).**ESI-MS**, *m/z*: 524.21 $[M-H]^-$.

Benzyl (2,2,3,3,4,4,5,5,6,6,7,7,7-tridecafluoroheptanoyl)-L-leucyl-L-phenylalanylglycinate (iii)

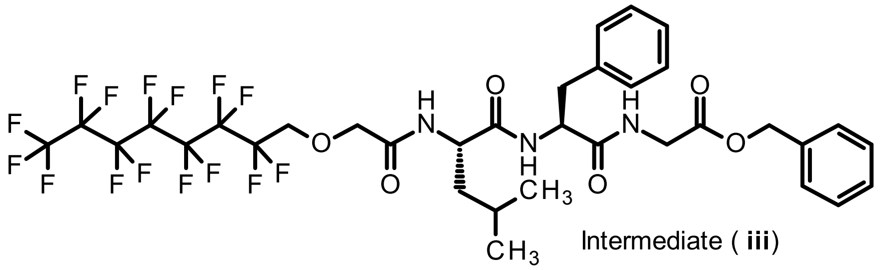

Intermediate (**2**) (58.3 mg, 0.11 mmol) was converted into the corresponding primary amine in quantitative yield following general procedure **b**. Deprotected (**2**) was reacted with perfluorinated acid **A** (71.5 mg, 0.16 mmol) according to general procedure $\mathbf{a_2}$. Intermediate **iii** was obtained (53.1 mg, 0.061 mmol, 55%, white solid) after a flash column chromatography on silica gel using a gradient of MeOH in DCM (R*f* = 0.88, DCM/MeOH 9:1).
^{1}**H NMR** (300 MHz, Chloroform-*d*), δ: 7.44 – 7.14 (m, 11H), 6.60 (d, *J* = 7.7 Hz, 1H), 6.52 (t, *J* = 5.5 Hz, 1H), 5.16 (s, 2H), 4.41 – 4.28 (m, 2H), 4.34 (s, 2H), 4.19 (t, *J* = 13.6 Hz, 2H), 3.16 (dd, *J* = 14.2, 6.5 Hz, 1H), 3.07 (dd, *J* = 14.2, 7.6 Hz, 1H), 1.75 – 1.38 (m, 3H), 0.90 (d, *J* = 6.0 Hz, 3H), 0.87 (d, *J* = 6.0 Hz, 3H); ^{19}**F NMR** (282 MHz, Chloroform-*d*), δ: -80.72 (t, J = 10.0 Hz, 3F), -119.45 – - 119.60 (m, 2F), -121.78 – -122.18 (m, 4F), -122.63 – -122.89 (m, 2F), -122.97 – - 123.42 (m, 2F), -125.94 – -126.19 (m, 2F); **ESI-MS**, *m/z*: 864.34 [M-H]$^-$.

(S)-N-((S)-1-((3-diazo-2-oxopropyl)amino)-1-oxo-3-phenylpropan-2-yl)-4-methyl-2-(2-((2,2,3,3,4,4,5,5,6,6,7,7,8,8,8-pentadecafluorooctyl)oxy)acetamido)pentanamide (1)

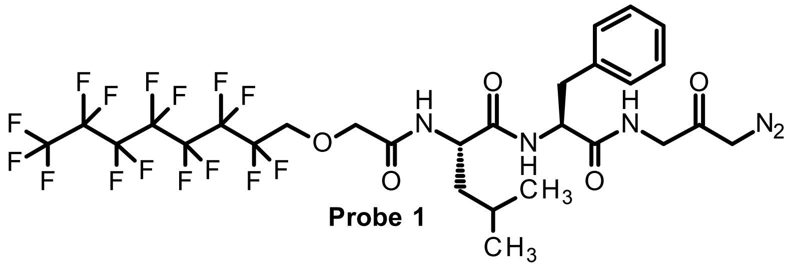

Intermediate (**3**) (126.1 mg, 0.15 mmol) was converted into the corresponding carboxylic acid in quantitative yield following general procedure **c**. According to general procedure **g**, deprotected (**3**) was reacted with a large excess of freshly synthesized (general procedure **f**) diazomethane in the presence of *i*BuOCOCl (0.22 mL, 0.17 mmol) as described in general procedure **h. Probe 1** was obtained as a yellow jelly-solid (104.1 mg, 0.13 mmol, 87%) after a digestion with diethyl ether. ^{1}**H NMR** (300 MHz, Chloroform-*d*), δ: 7.43 – 7.31 (m, 3H), 7.27 - 7.16 (m, 2H), 6.63 (d, *J* = 7.8 Hz, 1H), 6.51 (t, *J* = 5.4 Hz, 1H), 5.16-5.05 (m, 1H), 4.45 – 4.28 (m, 1H), 4.35 (s, 2H), 4.31 (bs, 1H), 4.15 (t, *J* = 13.6 Hz, 2H), 3.74 (bs, 2H), 3.18 (dd, *J* = 14.2, 6.5 Hz, 1H), 3.10 (dd, *J* = 14.2, 7.6 Hz, 1H), 1.77 – 1.40 (m, 3H), 0.95 (d, *J* = 6.0 Hz, 3H), 0.89 (d, *J* = 6.0 Hz, 3H); ^{19}**F NMR** (282 MHz, Chloroform-*d*), δ: -81.71 (t, J = 10.0 Hz, 3F), -118.47 – - 119.58 (m, 2F), -120.77 – -122.16 (m, 4F), -122.67 – -122.97 (m, 2F), -122.99 – - 123.47 (m, 2F), -125.95 – -126.21 (m, 2F); **ESI-MS**, *m/z*: 771.94 [M-H]$^-$.

- **Probe 2: Synthesis**

(2S,5S)-17,17,18,18,19,19,20,20,21,21,22,22,23,23,23-pentadecafluoro-2-(3-guanidinopropyl)-5-(4-hydroxybenzyl)-4,7,10,13-tetraoxo-15-oxa-3,6,9,12-tetraazatricosan-1-oic acid (2)

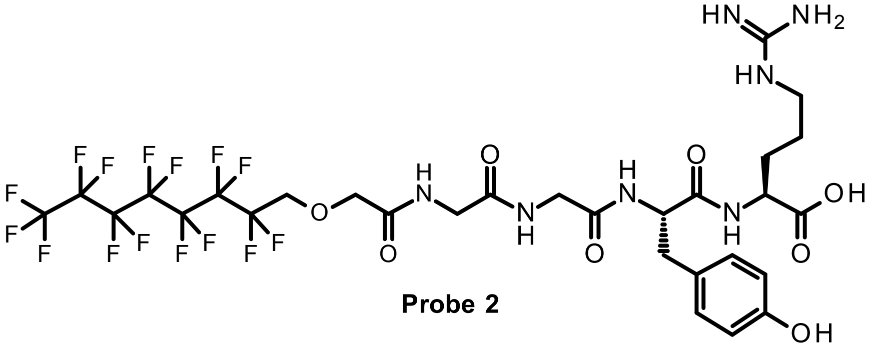

Probe 2

The GGYR portion of **probe 2** was prepared from 321.7 mg of 2-chlorotrityl chloride resin *via* an iterative cycle of peptide couplings (general procedures **j** and **i**) involving *N-Fmoc*-L-Gly-OH (2 x 460 mg), *N-Fmoc*-L-Tyr(O-*t*-Bu)-OH (710 mg) after the first coupling-capping step (general procedure **h**) done with *N-Fmoc*-L-Arg(Pbf)-OH (401 mg). After this, target compound was obtained by conducting a DIC-chemistry based coupling of GGYR with perfluorinated acid **A** (211 mg, 0.46 mmol) following general procedure **k**. **Probe 2** (29.8 mg) was obtained as a yellow solid after peptide cleavage and precipitation following general procedure **l**.

^{1}H NMR (300 MHz, Methan-d_4), δ: 7.08 (d, J = 8.1, 2H), 6.70 (d, J = 8.0, 2H), 4.55 – 4.51 (m, 2H), 4.44 – 4.41 (m, 2H), 4.33 – 4.23 (m, 5H), 3.95 – 3.74 (m, 5H), 3.24 – 3.18 (m, 3H), 3.11 – 3.04 (m, 2H), 2.91 – 2.83 (m, 2H), 1.99 – 1.95 (m, 2H), 1.77 – 1.62 (m, 3H); **^{19}F NMR** (282 MHz, Methan-d_4), δ: **-**76.96 (s, 3F), -82.37 (s, 2F), -121.104, 123.07 (m, 2F), -123.79, -124.35 (m, 4F), -124.352, - 127.35 (m, 4F); ESI-MS, ***m/z:*** 1783.76 [2M+1]$^+$
